# A descriptive study of Cambodian refugee infant feeding practices in the United States

**DOI:** 10.1186/1746-4358-3-2

**Published:** 2008-01-24

**Authors:** Becky Straub, Cathy Melvin, Miriam Labbok

**Affiliations:** 1 Department of Maternal and Child Health University of North Carolina at Chapel Hill, Chapel Hill, USA; 2 Center for Infant and Young Child Feeding and Care, Department of Maternal and Child Health, University of North Carolina at Chapel Hill, Chapel Hill, USA

## Abstract

**Background:**

The purpose of this exploratory study was to examine Cambodian refugee mothers' infant feeding beliefs, practices, and decision making regarding infant feeding in the U.S. and to explore if a culturally-specific breastfeeding program is appropriate for this community.

**Methods:**

A self-administered questionnaire and a 30 minute in-person interview were used to collect information from nine women. The audio-taped interviews were transcribed, answers compiled, and themes from each question identified.

**Results:**

All participants practiced either traditional Cambodian diet (pregnancy and postpartum diet including, *tnam sraa*, herbs mixed with either wine or tea), traditional Cambodian rituals (like *spung*, amodified sauna) or both, despite having lived in the U.S. for many years. All nine women initiated breastfeeding, however eight women introduced infant formula while in hospital. Perceived low milk supply and returning to work were the main reasons cited for partial breastfeeding and early cessation of breastfeeding.

**Conclusion:**

While causes of initiation of other foods are similar to those found in the U.S. as a whole, a culturally-specific Cambodian breastfeeding support program may help overcome some breastfeeding problems reported by Cambodian refugee mothers who have immigrated to the United States.

## Background

Immigrants in the U.S. generally have higher breastfeeding initiation and duration rates than U.S. born women; however, Southeast Asian groups, including Cambodian refugees, have been found to abandon their breastfeeding traditions soon after arriving in the U.S. [1-5]. Breastfeeding in Cambodia is nearly universal with 96% of children ever breastfed and an average duration of breastfeeding for 24 months [6]. However, the World Health Organization's (WHO) recommendation that infants be exclusively breastfed for the first six months is only practiced by about 12% of mothers [7,8]. A study of Cambodian infant feeding conducted from 1990–91 found that among 186 children born to Cambodian refugees in the U.S., only 18 were ever breastfed, with a mean duration of seven months although some of these children were only breastfed for a few days [9]. It is not known why Cambodian refugees changed their breastfeeding practices so quickly after arriving in the U.S., and/or if the traditional lack of exclusivity, diets and rituals around pregnancy and lactation are continuing, and if these customs influence infant feeding [6,8-10].

Cambodian refugees first came to the U.S. during the early to mid 1980s [9]. About 180,000 refugees were resettled in the U.S. and 18,000 were resettled in Canada [9]. The Cambodian refugees who came to the U.S. as children are now in their childbearing years.

This exploratory study examined Cambodian refugee mothers' infant feeding beliefs, practices, and decision making regarding infant feeding in the U.S. in order to explore if a culturally-specific breastfeeding program is appropriate for this community. The study focused on three main areas: knowledge and practice of traditional Cambodian pregnancy and postpartum customs as both a measure of acculturation and as an influence on their infant feeding practices; decision-making regarding infant feeding which includes: where and what types of infant feeding information they receive, who they trust; and the determinants of how they chose to feed their infant. This study explored whether these nine women, who have lived in the U.S. for most of their lives, have acculturated into mainstream U.S. practices, or whether Cambodian cultural practices are continuing on in the U.S. Furthermore, if their cultural practices have endured, which of them are healthy or potentially harmful practices.

## Methods

### Eligibility criteria

To be eligible for the study, subjects must have come to United States on a refugee visa (participants born in refugee camps qualified for the study), be the mother of a child between the ages of 6 months and 5 years old, have lived in the U.S. for at least 10 years, be at least 18 years of age and older, and speak English.

### Participant recruitment

The Cambodian Association of Illinois provided the primary researcher (BS) with names and phone numbers of women who met the eligibility criteria. This was a convenience sample and the results of this study cannot be directly generalized to the greater Cambodian community; however, it was designed to serve as formative research to inform future study. Thirteen women meeting eligibility criteria were contacted by telephone by the primary researcher and asked to participate. Ten women agreed to participate and, after giving their informed consent, were scheduled for an interview-one scheduled interview appointment was not kept. Reasons for declining an interview included: too busy working to set up a time to be interviewed (2 women), and self-report of not speaking English well (1 woman). Three interviews were held at the Cambodian Association of Illinois and six were at the participants' homes. Six of the interviews were conducted in the presence of young children, but none had significant interruptions or background noise. All of the women lived in Chicago or the surrounding suburbs. Interviews took place during the week of December 9–15, 2006. This study was reviewed and approved by the Institutional Review Board at the University of North Carolina at Chapel Hill.

### Survey instruments

We used a self-administered questionnaire and a 30 minute in-person interview consisting of 35 closed and open-ended questions. The primary researcher developed both survey instruments. The self-administered questionnaire had nine questions asking general demographic and behavioral information such as age, years of residence in the U.S, marital status, living with extended family, how often Khmer (Cambodian language) spoken at home, Women, Infants, and Children (WIC) program participation, educational level, and income. The interview consisted of 35 questions, six of which were modified from the WIC National Breastfeeding Promotion Project Sample Research Protocols questionnaire. Other questions were developed by the researcher based on information from past studies [4-6,9,10]. These questions included follow-up questions dependent upon the subject's response to an initial question. As an example of an embedded follow-up question about infant feeding, women were asked "Did your baby get formula while in the hospital?" If the respondent answered "yes", two follow-up questions were asked: Did you request for the baby to have formula or did someone else give formula to the baby? Why did you or the doctor/nurse decide to give the baby formula?

The questionnaire was pilot-tested with one Cambodian refugee mother in October 2006. The pilot test was to ensure that the level of English was appropriate, to assess whether the questions were interpreted the way the researcher intended, and to learn the correct pronunciation of Khmer words. None of the questions were significantly changed after the pilot test.

### Survey administration

To ensure that the subjects understood the written consent form, the researcher first read the consent form out loud and gave the participant time to read it to herself. After obtaining written consent, participants completed the self-administered questionnaire and the researcher sealed the questionnaire in an envelope to de-identify subjects. The envelopes were not opened until the end of the study. The researcher then received oral consent to begin audio-taping the subjects for the in-person interview. Interviews ranged from 13–42 minutes with a median time of 26 minutes. All in-person interviews were administered by the researcher in English.

### Analysis

Responses to the self-administered questionnaire were reviewed for completeness and a table of responses constructed. Participant characteristics were tabulated. Audio-tapes of the interviews with individual women were transcribed. Inconsistencies in responses were identified and resolved by asking the respondent to clarify her response. For example: to correctly assess problems, for example, with low milk supply, a series of related questions were asked: 1) Did you have any problems with breastfeeding? 2) Did your baby get formula in the hospital? 3) Who requested the formula? 4) Why? If the answer to the first question was "no" but "yes" to the second question, the third and fourth questions provided insight into the particular problem, i.e., low milk supply, that led to a request for infant formula. These inconsistencies were noted by the interviewer and clarified with the respondent during the interview. The interviewer then recorded the correct response to the initial question. Themes from each question and group of questions were identified and organized into six main areas: participant knowledge and practice of traditional Cambodian pregnancy, postpartum, breastfeeding diets and other Cambodian practices; infant feeding during the early months; infant feeding in the hospital; role of the family; low milk supply; and returning to work and weaning.

## Results

### Participant characteristics

Of the nine respondents, one was less than 28 years of age, two were 28–32 years, two were 33–37 years, and three were 38 years and older, and one did not respond. Two respondents reported living in the U.S. for 13–18 years and seven reported 19 or more years. Seven respondents were currently married, one divorced and one single. Three respondents did not finish high school, two were high school graduates, two had some college and two were college graduates. Four of nine respondents were living with an extended family member. All nine respondents reported speaking Khmer at home "most of the time". Eight respondents reported having ever been in the WIC program. All respondents were either close to or below 150% of the 2006 poverty level [11]. Respondents had a range of 1 to 4 children with an average of 3 children per mother. The average age of the youngest child was 2 years. All children born to respondents were born in the U.S. and in hospitals.

### Participant knowledge and practice of traditional Cambodian pregnancy, postpartum, breastfeeding diets and other Cambodian practices

All respondents knew of and participated in several traditional Cambodian diet and rituals surrounding postpartum and lactation. Eight women knew of and participated in some traditional dietary customs during pregnancy. Foods such as soup, *tnam sraa *(herbs mixed with either wine or tea), black pepper, and ginger were reported as foods that help women make enough milk. Foods for lactating women to avoid were spicy foods and 'stinky' foods such as fish sauce and *bahok *(fermented fish). These foods were said to give the baby diarrhea, cause an upset stomach, and make the baby susceptible to colds and fever. One woman said that part of her decision to wean was because she did not want to follow the breastfeeding diet any longer and worried about the health of her children.

"[Breastfeeding mothers should eat] a lot of soup. They say it makes the milk come out more...Sometimes the baby can get diarrhea if you don't eat a lot of soup."

All of the respondents had heard of, but did not participate in roasting, *ong klung*, which is a traditional postpartum practice in Cambodia that does not allow the baby to be breastfed for hours to days after the birth. None of the diets or rituals, such as s*pung *(modified sauna for the postpartum mother) and hot rock (warmed rock or brick wrapped in a towel and placed on the postpartum mother's stomach), were seemingly harmful to the baby nor mother and the baby did not need to be fed by an alternative care giver during any of the rituals.

### Infant feeding during the early months

During the first month, one respondent exclusively breastfed, five women gave about half breast milk and half infant formula, and three women gave mostly infant formula and some breast milk. Eight women thought that breast milk was healthier for babies than infant formula and one woman thought that breast milk and infant formula were equally healthy.

### Infant feeding in hospital

All respondents reported that they breastfed their baby while in hospital; however, eight of the nine women started giving infant formula while in hospital. All continued to do partial feeding (breastfeeding and infant formula) after discharge. One respondent exclusively breastfed in hospital and continued to exclusively breastfeed until stopping breastfeeding at six months. All received infant formula samples when discharged from hospital.

The eight women were asked who it was that had requested that the baby be given infant formula: a doctor/nurse, or did they request the infant formula, and why. Six women reported that the doctor or nurse gave them infant formula to feed the baby. The two women who self-requested infant formula while in the hospital did so because they felt that they did not have enough milk.

"I think the nurse brought it, the formula. And they asked me [if] I want to breastfeed and I said yes, she asked if I wanted formula and I said yes...Well, I was thinking that maybe I wasn't producing enough milk... I just didn't know if I had enough."

### Role of the family

All of the respondents reported that their family had helped them during the first month after their baby was born. Eight reported that they received and followed the advice from their mother or other relatives on what to feed their babies during the first year. For the first three months, five of the mothers reported that they were advised to breastfeed as much as they can. Almost all were advised to start rice soup as their baby's first solid food. Eight gave water to the baby before the introduction of solids, even though five of them had been advised by a doctor or other person to not give water. Three of the mothers reported that they were advised to give water by their mother or other relatives even if that contradicted what the doctor or WIC counselor told them. Relatives were also involved in preparing traditional foods and postpartum rituals for the women.

"My mom has experience, she has eight kids. And she raised really, really tough back home and so I believe her and we're alive even though we don't have that much food to eat and it's really hard to find food in that time during the Khmer Rouge, it was the killing fields...so I believe her more."

### Low milk supply

The eight women who started supplementing with infant formula while in hospital were asked why they decided to continue partial feeding during the first month. Six reported having a low milk supply with one or all of their children and that was the main reason they decided to supplement with infant formula. The women reported that they had a low milk supply for reasons such as: when they squeezed their breast, no milk came out; the other breast did not drip when feeding; the baby still cried after nursing; not much milk came out when she pumped; the baby lost weight; and the baby refused the breast and seemed to like infant formula better. Many of the women reported that they tried to boost their milk supply by eating certain foods such as: soups, pumpkin, and herbs; but these remedies did not work. Some had stated that they wanted to breastfeed, but they did not know why they did not make enough milk.

"They [mother or other relatives] said eat more soup to make more milk. They say, 'How come you don't have milk? If you like that in Cambodia your baby is going to starve to death.' Everything here we have, but I don't have enough milk...When I try to press out, it's not coming out. When I try to pump, it's not coming out, only when the baby suck."

### Returning to work and stopping breastfeeding

Seven of the women completely weaned all of their children from the breast at or before three months of age. The range varied from two weeks to twelve months. The one mother who exclusively breastfed weaned her infant to only solids over a period of two weeks when the child was approximately six months old. The mother who breastfed for twelve months, combined infant formula feeding and breastfeeding the entire time. She was the only respondent who continued breastfeeding after returning to work.

All the women who practiced partial breastfeeding reported that they had planned while they were pregnant to return to work or school around two to three months postpartum, which they did. Five of the women stated that returning to work affected their decision to do partial bresatfeeding. One woman practiced partial breastfeeding so that the baby would get used to the infant formula when she went back to work. Some of the respondents mentioned that their babies refused the breast after a few weeks or months and since they were returning to work or had already started working, they went along with the weaning and switched to infant formula, exclusively. Several of the mothers mentioned that they tried to express milk by pumping; however, they did not get very much milk and stopped. Others knew that they would not pump when returning to work and would switch over completely to infant formula within two or three months after the birth.

"She [my mother] say when she have a baby she's breastfeeding for a year or two year. But in the United States I cannot do like her because we work and we do not have time."

## Discussion

Immigrants in the United States generally have higher breastfeeding initiation rates than U.S. born women [1-3]. A study of Hispanic and black women and their country of origin found that foreign born black and Hispanic women were more likely to intend to only breastfeed than U.S. born women (42% vs 24%) [2]. Researchers have also found that more acculturated immigrants and years of residence in the U.S. negatively impacts breastfeeding initiation and duration [12-14].

Southeast Asian groups have been found to abandon their breastfeeding traditions soon after arriving in the U.S. [4,5]. A study of 134 Southeast Asian mothers in Stockton, CA found that 97% of the women breastfed their last infant born in Asia, while only 22% breastfed their last infant born in the U.S. [4]. Only 4% of the pregnant women in the study intended to breastfeed [4]. A study of Hmong and Vietnamese mothers in California found a low rate of breastfeeding among the women [4]. Only 8% of the Hmong women and 5% of the Vietnamese women breastfed their youngest child [4]. A study of Cambodian refugee breastfeeding practices found that there was almost a 100% breastfeeding initiation rate among children born in Cambodia or in refugee camps, but only 18 out of 186 children born in the U.S. were ever breastfed [9].

### Breastfeeding: new culture and new challenges

Unlike previous studies of Southeast Asian immigrants and Cambodian refugees, all the women in this study initiated breastfeeding; although most did not do so exclusively [5,6,9]. None of the women restricted breastfeeding during the practice of traditional Cambodian postpartum diet or rituals. Eight of the nine women reported that breastfeeding is healthier for babies and one respondent reported that both breastfeeding and infant formula feeding are equally as healthy. Some of the women stated that breastfeeding is a part of Cambodian culture and traditions. If this group is representative of the general point of view of today's Cambodian refugee population, the overwhelmingly positive view of breastfeeding shows that perception is not a barrier that would need to be overcome in supporting Cambodian women to breastfeed.

However, the study has identified areas that are common to the U.S. population in general and may be addressed in educational and support services and areas that need future research to help avoid some of the problems that these respondents had with breastfeeding such as: knowledge of colostrum; importance of exclusive breastfeeding for six months as recommended by the WHO; supply and demand in milk production; signs of low milk supply and ways to correct it; and options for returning to work.

A family-centered breastfeeding program may be an appropriate way to support Cambodian refugees in particular to breastfeed. The respondents' mothers or other relatives provided help after the baby was born, prepared traditional foods and rituals for the mother, gave infant feeding advice, and about half of the women were living with an extended family member. In some instances, respondents followed the advice of their relatives even if it contradicted what the doctor had told them. It is unknown to what extent their mother or other relative's knowledge of breastfeeding affected the respondents ability to breastfeed; however, it is clear that the family is involved in providing support and infant feeding advice after the birth. This could also serve as a venue for introducing new behaviors, such as exclusive breastfeeding, which was not the norm traditionally in Cambodia.

All the women in the study stated that they did not have any milk or enough milk after the baby was born. There was a real concern over what their babies were going to eat until the milk came in. Questions regarding knowledge of colostrum would be useful in future research as the researcher did not ask directly about colostrum and none of the women mentioned the word or concept of colostrum. Educating the family and grandmother about colostrum is important because the traditional postpartum practice of roasting, *ong klung*, is widely practiced in Cambodia. This practice does not allow the mother to breastfeed the baby for up to a few days after the birth, which results in the baby not receiving colostrum [8]. While none of the women did *ong klung*, the grandmothers who did *ong klung *in Cambodia may not know about colostrum nor the importance of giving the baby colostrum since they may not have breastfed during the first few days postpartum. Educating the family about the importance of colostrum may prevent the early introduction of infant formula and bottles, increase frequency and exclusivity of breastfeeding, and subsequently allay concerns with low milk supply.

Perceived low milk supply was cited by six of the nine women as the main reason they started giving infant formula. Most of the signs that the women perceived as signs of low supply are not, in fact, indicative of a low milk supply. Some of the women ate traditional foods to help boost their milk supply; the traditional breastfeeding diet is healthy but the concern is that these foods were mentioned as the only method that the women tried to boost their milk supply. None mentioned either eliminating the bottle, or breastfeeding more frequently, as strategies to increase their supply. The early introduction of bottles may have disrupted their supply, and nipple confusion may have distracted the babies from learning how to breastfeed effectively. Education about milk production, supply and demand, signs of sufficient and insufficient milk supply, and limiting bottles and pacifiers in the early weeks may help prevent low milk supply and the unnecessary supplementation of infant formula for women who are actually producing enough milk.

Providing Cambodian women with information and support on different breastfeeding options may help increase exclusive breastfeeding and breastfeeding continuation after returning to work. Eight of the nine women returned to work or school within three months of giving birth. Some of the women wanted to continue breastfeeding after returning to work and some stated that they knew they would use only infant formula after returning to work. Most of the women stated that returning to work was one reason they introduced infant formula in hospital. The exclusivity and duration of breastfeeding has been found to have the greatest health effects for the baby [15,16].

A culturally sensitive breastfeeding program may be able to give the women more options on how to feed their babies in the early months. First, if women do want to continue to breastfeed after returning to work, mothers could be informed about the different types of pumps, hand expression, storage of human milk, and how to keep their milk supply up when returning to work. Most of the mothers in this study reported being on the WIC program which may make them eligible for a free breast pump and free infant formula [17]. Awareness of the WIC supplementary food package for breastfeeding mothers might also contribute to their decision to choose breastfeeding instead of the free infant formula. Mothers can be given support and suggestions on how to negotiate with an employer about the time and space needed to pump. A second option would be for mothers to do partial breastfeeding or reverse rhythm feeding (i.e., increased frequency at night, and fewer supplemental feeding in the day) when returning to work. This would allow the mother to continue to breastfeed her baby and not have to pump or not pump as frequently while working. A third option for women who do not want to continue breastfeeding after returning to work is to educate them on the benefits of exclusive breastfeeding during the early months. They can exclusively breastfeed for at least the two to three weeks before returning to work and gradually wean the baby to infant formula thereafter.

## Conclusion

The Cambodian refugees who participated in this study practiced many Cambodian traditions around pregnancy and the postpartum period despite having lived in the U.S. for many years. All the women reported receiving help from their mothers or other family members during the first month after their baby was born and most of the women are receiving and following infant feeding advice from their mothers who may not have knowledge of some of the challenges that women in the U.S. encounter when they breastfeed: infant formula availability, bottles, pacifiers, and returning to work.

A family-centered breastfeeding program may be the best option for this community to encourage the continuation of the many positive dietary and postpartum rituals that the women continue in the U.S. and to supplement their breastfeeding knowledge regarding: colostrum, supply and demand in milk production, importance of exclusive breastfeeding, signs of low milk supply and ways to correct it, and options for returning to work. This formative research may contribute to future research, and raises questions such as: what breastfeeding support and information is being taught from Cambodian grandmothers to their daughters that would better inform, or what breastfeeding information needs to be supplemented. The limitations of this study are its small sample size including only women who had been in the U.S. for many years and who spoke English. While many of the problems reported in this study are not specific to this population, a culturally-specific Cambodian breastfeeding program may help overcome some breastfeeding problems that Cambodian refugee mothers are experiencing in the U.S.

## Competing interests

The author(s) declare that they have no competing interests.

## Authors' contributions

BS designed the study, recruited and interviewed participants, analyzed data, and drafted the manuscript. CM and ML supervised all aspects of the study and provided critical revisions of the manuscript.

## References

[B1] Celi AC, Rich-Edwards JW, Richardson MK, Kleinman KP, Gillman MW (2005). Immigration, race/ethnicity, and social and economic factors as predictors of breastfeeding initiation. Arch Pediatr Adolesc Med.

[B2] Bonuck KA, Freeman K, Trombley M (2005). Country of origin and race/ethnicity: impact on breastfeeding intentions. J Hum Lact.

[B3] Gibson-Davis CM, Brooks-Gunn J (2006). Couples' immigration status and ethnicity as determinants of breastfeeding. Am J Public Health.

[B4] Reeves Tuttle C, Dewey KG (1994). Determinants of infant feeding choices among Southeast Asian immigrants in northern California. J Am Diet Assoc.

[B5] Institute of Medicine (1991). Nutrition During Lactation.

[B6] National Institute of Statistics, Directorate General for Health [Cambodia], and ORC Macro (2001). Cambodia Demographic and Health Survey 2000 Maryland.

[B7] Global Strategy for Infant and Young Child Feeding. http://www.who.int/nutrition/publications/gs_infant_feeding_text_eng.pdf.

[B8] Childinfo. http://www.childinfo.org/areas/breastfeeding/countrydata.php.

[B9] Rasbridge LA, Kulig JC (1995). Infant feeding among Cambodian refugees. MCN AM J Matern Child.

[B10] Kemp C (1985). Cambodian refugee health care beliefs and practices. J Comm Health Nurs.

[B11] Federal TRIO Programs 2006 Annual Low Income Levels. http://www.ed.gov/about/offices/list/ope/trio/2005-low-income.html.

[B12] Gibson MV, Diaz VA, Mainous AG, Geesey ME (2005). Prevalence of breastfeeding and acculturation in Hispanics: results from NHANES 1999–2000 study. Birth.

[B13] Rassin DK, Markides KS, Baranowski T, Richardson CJ, Mikrut WD, Bee DE (1994). Acculturation and the initiation of breastfeeding. J Clin Epidemiol.

[B14] Guendelman S, Siega-Riz AM (2002). Infant feeding practices and maternal dietary intake among Latino immigrants in California. J Immigr Health.

[B15] Ball TM, Wright AL (1999). Health care costs of formula-feeding in the first year of life. Pediatrics.

[B16] Anderson JW, Johnstone BM, Remley DT (1999). Breast-feeding and cognitive development: a meta-analysis. Am J Clin Nutr.

[B17] WIC- Breastfeeding promotion and support. http://www.fns.usda.gov/wic/Breastfeeding/breastfeedingmainpage.HTM.

